# Modifying the school determinants of children’s health

**DOI:** 10.1177/01410768211051718

**Published:** 2021-12-02

**Authors:** Chloe Lowry, Ingrid Stegeman, Franz Rauch, Anant Jani

**Affiliations:** 1 UCL Institute of Education, University College London, London WC1H 0AL, UK; 2 EuroHealthNet, Brussels, Belgium; 3 27256 University of Klagenfurt, Klagenfurt am Woerthersee, Austria; 4 Oxford Martin School, University of Oxford, Oxford OX1 3BD, UK; 5 Heidelberg Institute of Global Health, University of Heidelberg, 672 69120 Heidelberg, Germany

Our living environments have a significant impact on our health: the ‘social determinants of health’ account for 80–90% of health outcomes.^1^ For adults, these determinants are spread across a range of contexts, but most children's lives are dominated by only two environments: home and school.

Schools are where children eat, play, and exercise, as well as learn. They are the communities in which children make friends or face bullying, learn norms of healthy behaviour or dangerous risk-taking, connect with trusted adults outside the family or reject authority and face discipline.

Given there are innumerably more families than schools, the school environment is eminently more modifiable than the family. This commentary, which is part of a series exploring the nexus between the education and health sectors, explores how the school determinants of children’s health can be modified to improve population outcomes and outlines a proposal for the next generation of health promoting schools.

## The school effect on children’s health

**Figure 1.**
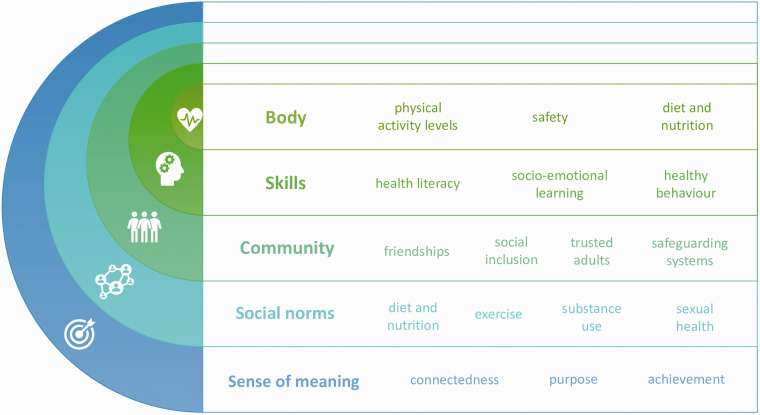
How a child's health can be impacted by their school.

Schools have a significant effect on pupils’ intellectual, spiritual, moral, social and cultural development. There is also a strong bi-directional relationship between education and wellbeing as well as physical and mental health: health promotion initiatives improve academic outcomes, and educational achievement improves health throughout the lifespan. Yet formal education is only one of the mechanisms through which schools ‘determine’ health. A child’s long-term physical and mental health is impacted by their school in a myriad of direct and indirect ways (Figure 1).

This impact varies between schools. Differences in school climate, policies and ethos have been found to explain up to 40% of the variance in children’s substance misuse, and children’s physical activity levels are linked to the Physical Education provided at their school.^2^ Across Europe and North America, children who perceive their school as supportive are more likely to engage in healthy behaviours and have better health outcomes.^3^

Children’s sense that their teachers and classmates care about them, or ‘school connectedness’, has lifelong impact. In a longitudinal study of 36,000 adolescents, this was the strongest protective factor for decreasing substance misuse, violence, early sexual initiation and risk of injury, and second only to family connectedness in protecting against emotional distress, disordered eating and suicide.^4^ While much of the variance in connectedness depends on individual circumstances, over a quarter is explained by school-level variables, with the priority placed on pastoral care identified as one of the most significant (in the UK context, pastoral care refers to holistic support for children's welfare and is not strictly linked to any religious teaching).^5^

Schools have a particularly significant role for disadvantaged children and can mitigate the risks they face in other areas of their lives. Vulnerable youth who feel connected to their school have fewer emotional and behavioural problems than those who don't, and the positive impact on self-esteem and suicidal ideation is as significant as family connectedness.^6^

Although many schools seek to promote holistic child development through pastoral care, much is still unknown about the impact of different school structures, policies and systems (e.g. assessment, pastoral, disciplinary) on these factors.^5^ The lifelong significance of health and health-related behaviour in childhood means understanding and improving the school determinants of health must be a public health priority.

## The Health Promoting School

Since 1995, the World Health Organization (WHO) has advocated the Health Promoting School (HPS) model as a way of modifying the school determinants of health. An HPS ‘constantly strengthens its capacity as a healthy setting for living, learning, and working’.^7^

The model promotes the health of both pupils and staff through six evidence-based mechanisms:

- Healthy school policies (e.g., smoke-free, healthy food)
- Physical school environment (e.g., safety standards, space for physical activity)
- Social school environment (e.g., inclusive school ethos, anti-bullying)
- Health education and skills (e.g., curriculum time, teacher training)
- Links with parents and community (e.g., consulting parents, collaborating with community groups)
- Access to school health resources (e.g., school counsellor, first aid)^7^

While the traditional approach to school health promotion reduced schools to convenient venues for knowledge-based interventions, research suggests that holistic complex, multifactorial initiatives that bridge the domains of curriculum, school environment and community are more likely to suceed.^8^ A Cochrane systematic review of HPS initiatives found positive impacts on children’s physical fitness, activity levels, diet, smoking and experience of bullying.^9^

However, research also shows that modifying school determinants is not always straightforward. In the Cochrane review, only a few eligible studies examined the impact on substance misuse and mental health; those that did found no evidence of effectiveness.^9^ Other research, however, suggests HPS can improve these outcomes: one synthesis of systematic reviews found that HPS initiatives focussed on mental health were among the most effective,^8^ and a meta-analysis demonstrated the effectiveness of substance use interventions directed at the school context.^10^

The HPS model has merit, but reliably modifying the school determinants of health has proved challenging.

## The challenge of creating a healthy school

Modifying the school determinants of health is a process that requires multi-stakeholder investment and engagement. Globally, the WHO has identified significant barriers to effective HPS implementation and emphasises the need for structural change, including:

- Better data collection systems for monitoring and evaluation
- Long-term partnerships between stakeholders at all levels, including national health and education departments
- Sustainable funding and long-term financing plans
- Improving the quality of teaching and time dedicated to health education
- Institutionalised human resource development, including pre- and in-service teacher training^11^

Many of these barriers were visible in the UK's own HPS initiative, the National Healthy Schools Scheme.

### National Healthy Schools Scheme

The scheme began in 1999 and ran for a decade. All schools were encouraged to achieve the Healthy School award by fulfilling the criteria, and uptake was high. A national comparison of award and non-award schools found that pupils at secondary award schools were significantly more likely to engage in healthy behaviour, and that this had improved over time.^12^ However, no overall difference was found in primary schools, and a subsequent study of 152 schools found that achieving the award had no significant impact on healthy behaviour.^13^

Analyses of the initiative identified three key weaknesses. First, the approach to monitoring and evaluation was not consistent across sites. Local authorities defined Healthy School status differently,^12^ and schools self-validated their status by choosing evidence from a disparate range of metrics. For example, suggested outcome metrics for health education ranged from ‘staff report they enjoy teaching [it]' to ‘there is a reduction in teenage pregnancies'.^14^ Programme co-ordinators raised concerns about self-validation and the quality assurance process.^15^

Second, schools were not consistently provided with the training and support required for meaningful change. Whether there was funding available to train teachers varied widely, depending on whether the local programme was able to secure it elsewhere or persuade trainers, like primary care trusts, not to charge. The support offered by co-ordinators also varied, and sometimes a lack of understanding of the local context limited their value.^15^

Finally, the depth of engagement required to change school culture appears to have been missing. Schools often already fulfilled the majority of the scheme's criteria, and only 20% said that implementing it had significantly changed their practice.^13^ The scheme's theoretical model also appears insufficiently complex to reflect how institutions impact behaviour^9^: e.g. an updated food policy is hypothesised to prompt pupils to want a healthy packed lunch due to improved knowledge of nutrition.^13^

## Embracing the challenge

Initiatives that attempt to modify the school determinants of health must be prepared to embrace the challenge. Interventions that have invested in appropriate infrastructure have been met with success.

### Hong Kong Healthy Schools Award

Hong Kong sustained and scaled up its HPS movement over the course of two decades. Thorough groundwork was laid at the start: a professional diploma in health promotion and health education was created for school staff and a new professional association brought together professionals from different backgrounds.^16^ The award evaluation process was thorough, and results were impressive, with pupils at award schools demonstrating significant improvement in a range of health outcomes, and academic attainment, compared to non-award schools. Thorough data collection allowed researchers to assess which school-level changes had the most impact; training teachers and supporting their wellbeing featured prominently.^17^

### South West Healthy Schools Plus programme

The UK found success in a pilot follow-up programme for schools in deprived areas that had achieved the national award. Local co-ordinators worked with schools, using data to identify three areas for intervention (based on school priorities, local health priorities, and the needs of vulnerable children) and assessing the impact. Almost 4000 interventions took place in over 1000 schools and the healthy behaviours targeted increased by 250% on average. The support of the local HPS co-ordinators and the systematic use of data were considered key to this success.^18^

With investment and support, HPS can successfully modify the school determinants of children's health. However, more research is needed to establish what works, for whom, and in which circumstances.^9^ The proposal for the next generation of HPS below aims to both learn the lessons of past initiatives and break new ground in generating the evidence base for a healthier future.

## The next generation of health promoting schools

The WHO's vision of an HPS as constantly strengthening its capacity as a healthy setting for living, learning and working is ambitious. Going beyond encouraging schools to achieve a national standard, it envisages the whole school engaged in an ongoing process of continuous improvement. Recent technological advances make achieving this ambition possible.

The transformation in our capacity to collect and analyse data since the WHO first conceived of HPS opens up the opportunity for an individualised and responsive approach to modifying the school determinants of health. Schools could use anonymised data on the health and wellbeing of their pupils and staff to inform interventions on an ongoing basis, while also linking this data with healthcare systems. Collecting this strategically on a wider scale would fill in the gaps of how school-level factors impact health, enabling the development of a blueprint of a healthy school. Evaluating interventions would illuminate what works, for whom, and in which circumstances, leading to a toolbox of increasingly effective interventions.

Encouragingly, an initiative showcasing some of these features is beginning across secondary schools in Greater Manchester, a region of significant socio-economic deprivation. Pupils will complete annual wellbeing assessments covering key areas including mental health, healthy behaviour and social support; the Child Outcomes Research Consortium will support schools to understand the data and improve provision.^19^

For the WHO's vision to become a reality, however, more is required than pupil data. A whole-school approach cannot be realised without meaningful engagement with the creators and custodians of the school environment: the teacher.

Worryingly, teachers are consistently found to experience higher work-related stress and poorer mental health than other occupations. Despite the implications for their long-term health, and evidence that teachers' mental health and wellbeing impacts the mental health, wellbeing, and educational outcomes of their pupils, research into supporting teacher wellbeing is scarce.^20^

In addition, teachers internationally do not receive sufficient training as health promoters or for specific school-based initiatives, despite their central role in children's health. Australia broke the mould by developing health promotion training for teachers at scale over the last two decades, and is currently offering all educators free accredited training through the ‘Be You' initiative.^21^

**Figure 2.**
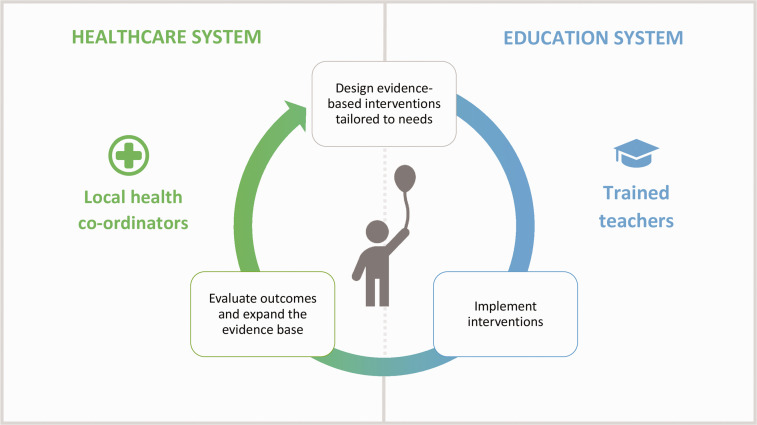
Creating the next generation of health promoting schools in the UK.

Learning from past successes and failures, we propose a new national initiative to enable the UK and other countries to take a holistic approach to children's health and develop the next generation of HPS, through three key mechanisms (Figure 2):

- Delivering high-quality, mixed-mode, pre-service and in-service training for all teachers in promoting child and/or adolescent health, tailored to their specific roles (in school leadership, teaching health education, delivering pastoral care, etc.).
- Establishing national, regional, and local networks of health co-ordinators who work collaboratively with schools and monitor the health and wellbeing of pupils and staff, and
- Using data on inputs, outputs, and outcomes to generate learning loops that support the design and implementation of interventions tailored to local and school needs.

Ambitious ends require ambitious means. The potential benefits to the health of the children and teachers concerned, and to future populations, of investing in a process that works iteratively to create a healthier education system are significant. The personalised data-led approach ensures that interventions are optimised for the context and are recognised as relevant by schools, improving buy-in. Collaboration between health co-ordinators and teachers would begin to break down the structural, institutional and perceptual barriers that restrict us to addressing health independently of the factors that influence it. The data generated would provide unprecedented insight into the school determinants of health and how they can be successfully modified, and the infrastructure created in the process would enable an agile and effective response to the emerging needs of children as we move beyond the COVID-19 pandemic.
